# Is there relationship between SARS-CoV-2 and the complement C3 and C4?

**DOI:** 10.3906/sag-2004-336

**Published:** 2020-06-23

**Authors:** Hamad DHEİR, Savaş SİPAHİ, Selçuk YAYLACI, Mehmet KÖROĞLU, Ali Fuat ERDEM, Oğuz KARABAY

**Affiliations:** 1 Division of Nephrology, Faculty of Medicine, Sakarya University, Sakarya Turkey; 2 Department of Internal Medicine, Faculty of Medicine, Sakarya University, Sakarya Turkey; 3 Department of Microbiology, Faculty of Medicine, Sakarya University, Sakarya Turkey; 4 Department of Anesthesiology, Faculty of Medicine, Sakarya University, Sakarya Turkey; 5 Department of Infectious Diseases, Faculty of Medicine, Sakarya University, Sakarya Turkey

## Dear Editor

The new coronavirus (COVID-19) pandemic continues to affect human health worldwide seriously. The disease is spread mainly via large droplets. A lot of cases are asymptomatic, while the others are symptomatic. The main symptoms are fever, cough, shortness of breath, while anosmia, conjunctivitis, and gastroenteritis are less common. Until an effective vaccine or treatment is found, the most important means of prevention remains to be social isolation. COVID-19 patients showed lymphopenia, abnormal respiratory findings, and increased levels of plasma proinflammatory cytokines u28b3. SARS-CoV-2 virus is a respiratory system attacking agent causing severe pneumonia and other vital systemic features including cardiac injury u28b6. Furthermore, hematologic and coagulation abnormalities, such as elevated ferritin, D-Dimer, prolongation of prothrombin time/activated partial thromboplastin time, and thrombocytopenia are common among these patients u28b7.

There remain a considerable number of questions that need to be answered to be able to understand this virus. While the complement system is a critical component of the innate immune system, uncontrolled activation of complements can cause severe diseases like thrombotic microangiopathy. 

Of note, hypocomplementemia was described in various viral infections like parvovirus B19, Ebola virus, and hepatitis C infections u28b8u28b9. Recently, it was shown that the complement activation contributes to severe acute respiratory syndrome coronavirus (SARS-CoV) pathogenesis in mice u28ba. Also, a case-control study showed that lower serum mannose-binding lectin (MBL) in 353 patients with SARS-CoV were determined 19–23 days after the onset of disease. They found that the median serum level of MBL in these patients (0.733 [IQR, 0.263–1.796] mg/mL) was significantly lower than that in 1167 control individuals (1.369 [IQR, 0.572–2.598] mg/mL). These findings suggested that MBL-deficient patients were more susceptible to SARS-CoV, and they provide insight into the possible mechanisms of the innate immune response to SARS-CoV infection u28bb. To our knowledge, there is no published data yet neither in vitro nor in vivo demonstrating the relationship between COVID-19 and C3 and C4 levels. In our center, we retrospectively analyzed the association of hypocomplementemia and COVID-19 infection. 

We retrospectively analyzed C3 and C4 levels of fifty-seven COVID-19–positive patients, including 29 intubated intensive care unit (ICU) patients and 28 non-ICU ward patients. Serum complement levels were measured at admission. The median value of C3 and C4 levels in ICU patients was (1.15 [IQR 0.91–1.39] g/L) and (0.24 [IQR 0.12–0.34] g/L), whereas in non-ICU patients there were (1.48 IQR [1.24–1.72] g/L) and (0.30 g/L [IQR 0.24–0.40]), respectively (P > 0.05). In terms of tendency to decreased C3 levels, the difference between two groups was insignificant. 

According to our findings, there was no significant difference in terms of C3 and C4 levels in both ICU and non-ICU COVID-19 patients. For this reason, we think that C3 and C4 levels cannot be used to show disease activation. Therefore, we need more clinical studies to confirm our outcomes.
